# Test-Retest Reliability of Acoustic Emission Sensing of the Knee during Physical Tasks

**DOI:** 10.3390/s22239027

**Published:** 2022-11-22

**Authors:** Liudmila Khokhlova, Dimitrios-Sokratis Komaris, Salvatore Tedesco, Brendan O’Flynn

**Affiliations:** 1 Insight Centre for Data Analytics, Tyndall National Institute, University College Cork, T12 R5CP Cork, Ireland; 2 Faculty of Science and Engineering, School of Engineering and the Built Environment, Anglia Ruskin University, Bishop Hall Ln, Chelmsford CM1 1SQ, UK

**Keywords:** joint sound, measurement properties, on-body sensor monitoring, joint health, repeatability

## Abstract

Acoustic emission (AE) sensing is an increasingly researched topic in the context of orthopedics and has a potentially high diagnostic value in the non-invasive assessment of joint disorders, such as osteoarthritis and implant loosening. However, a high level of reliability associated with the technology is necessary to make it appropriate for use as a clinical tool. This paper presents a test-retest and intrasession reliability evaluation of AE measurements of the knee during physical tasks: cycling, knee lifts and single-leg squats. Three sessions, each involving eight healthy volunteers were conducted. For the cycling activity, ICCs ranged from 0.538 to 0.901, while the knee lifts and single-leg squats showed poor reliability (ICC < 0.5). Intrasession ICCs ranged from 0.903 to 0.984 for cycling and from 0.600 to 0.901 for the other tasks. The results of this study show that movement consistency across multiple recordings and minimizing the influence of motion artifacts are essential for higher test reliability. It was shown that motion artifact resistant sensor mounting and the use of baseline movements to assess sensor attachment can improve the sensing reliability of AE techniques. Moreover, constrained movements, specifically cycling, show better inter- and intrasession reliability than unconstrained exercises.

## 1. Introduction

The knee is one of the most complicated and highly loaded joints in the human body. It is particularly vulnerable to injuries and degenerative disorders, such as knee osteoarthritis (OA). According to a US study conducted by Murphy et al. [1], the lifetime risk of developing knee osteoarthritis has been estimated at 45%. Currently, surgical methods such as joint arthroplasty are commonly used to restore the joint’s function and reduce pain in cases of severe OA. The estimated mortality-adjusted lifetime risk of undergoing total knee replacement surgery is 10.8% for women and 8.1% for men at the age of 50, based on the results provided by the UK general practice research database [2].

Population ageing and the prevalence of obesity [3] can only add to the burden of the incidence rate of OA. This, coupled with the increasing access of the general population to healthcare resources and technological advances, means that the number of total joint replacements is expected to increase dramatically in the coming decades [4,5]. This prognosis is further supported by the growing numbers of total knee arthroplasty (TKA) surgeries reported in several countries (the UK, New Zealand, the USA, Austria and Sweden) [6].

Current clinical standards in the non-invasive monitoring of joints include imaging technologies (e.g., X-radiography, MRI and CT), physical examination (e.g., range of motion tests), functional tests (e.g., 6-min walking test), and patient-reported pain/outcome questionnaires (e.g., WOMAC, KOOS and OKS). Imaging techniques are considered to be expensive and are only available at clinical sites; functional tests and physical evaluations are highly dependent on the physician’s experience; and personal questionnaires are inherently subjective and not adequately sensitive. Moreover, such methods are not always suited for potential telemedicine applications or appropriate for at-home rehabilitation; thus, alternative solutions aiming to improve the quality of post-surgery care are currently being investigated [7,8]. With the current advances in the area of wearable technologies and personalized healthcare, the development of non-invasive methods for joint status assessment that are able to provide quantitative measurements and track treatment/rehabilitation processes has made significant progress recently [9].

Acoustic emission (AE) monitoring is widely used in the non-destructive testing of structures and machinery and has recently gained renewed attention in orthopedic applications [10]. An AE can be defined as a transient elastic wave generated by a rapid release of energy within the investigated material or object [11]. AE monitoring is used to detect small-scale damage within materials during structural proof tests, and typical applications include the detection of pores, leaks, fatigue, corrosion, fiber cracks, delamination and loose parts, as well as monitoring processes, such as deforming or welding. In the context of orthopedics, AE monitoring was initially adopted for destructive and ex-vivo tests, such as the investigation of bone structure and crack detection [12], while more recently, the research focus has shifted to the non-invasive diagnostics of bone and cartilage conditions [10]. Age-related deterioration [13,14], osteoarthritis [15,16,17], and past injuries [18,19] are currently assessed via AE event detection, and results have been found to show promise in a number of studies in recent years.

**Figure 1:**
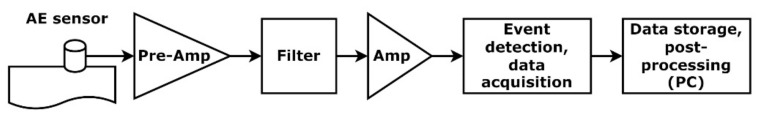
AE monitoring system: flow-chart.

The workflow of a typical AE monitoring system is depicted in Figure 1 (top). Initially, a piezoelectric sensor converts dynamic surface motion at the transducer interface into electric signals. The signal is then amplified, firstly by the pre-amplifier embedded into the AE sensor itself or by a separate circuit, and subsequently by the main amplifier within the data acquisition unit. After amplification, signals can be filtered and then processed.

**Figure 2:**
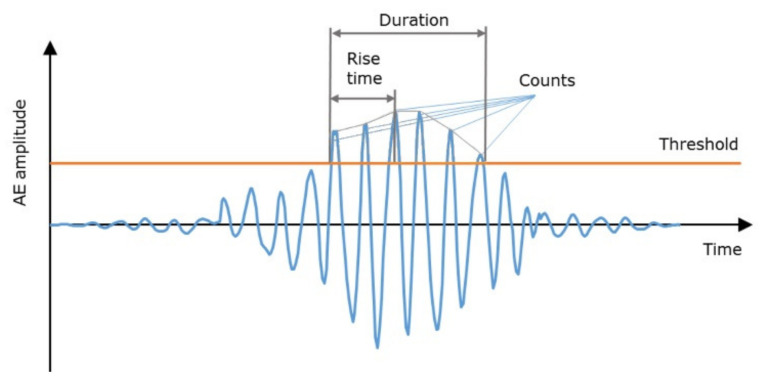
AE event (hit) parameters.

Signal processing in AE monitoring usually involves acoustic event (hit) detection. In this regard, an event is usually detected when the AE signal exceeds a preset or a floating amplitude threshold. Hit detection is typically defined by the following parameters (Figure 2): amplitude threshold; hit definition time (HDT), which specifies the maximum time between threshold crossings; hit lockout time (HLT), which is defined by the time that must pass after a hit has been detected and before the next one occurs and can be detected again; and peak definition time (PDT), which is determined by the time from hit detection to peak [20]. To further detect and analyze a defect or a process, several characteristics of the detected hits can then be extracted and used. Such parameters may include the number of hits, the hit duration, the rise time and the number of excursions over the threshold (counts).

While multiple studies in joint assessment have shown positive results in the application of AE monitoring for distinguishing deteriorative joint conditions, the majority of the research in the field consists of pilot and/or small-scale validation or feasibility studies. According to the results of the scoping review by Khokhlova et al. [10], 16 out of the 24 studies on joint assessment had fewer than ten participants/specimens per researched group. Moreover, data recording and processing techniques varied widely between studies, and the lack of standardized procedures, reliability assessments, and in particular test-retest (inter-day) evaluations of AE monitoring was evident. Considering practical implications such as unreliable measurement of the effect of acute treatment, long-term therapies, or disease progression, the overall utility of AE monitoring is dependent on the method’s reliability over repeated measurements both within and between sessions (inter-day, test-retest).

The first work to investigate the inter-day reliability of knee sound monitoring was recently presented by Kalo et al. [21]. Authors evaluated recordings of knee sounds (at 100–300 Hz) from two locations (medial tibial plateau and the center of the patella) during sit-to-stand movements. In that study, MEMS microphones were attached to the skin with double-sided tape. The amplitude and median power frequency of the recordings were found to be highly reproducible within a single session: intraclass correlation coefficients (ICCs) ranged from 0.85 to 0.95 for the tibia and from 0.73 to 0.87 for the patella; however, inter-day ICCs were inconsistent (0.24 to 0.33 for the tibia and from 0 to 0.82 for the patella). The authors suggested that replacing the microphones may lower inter-day reliability, and that measurements may benefit from internal standardization by means of a reference measurement, for example, a zero-load (passive movement) recording.

More positive results were acquired in [22] in the reliability assessment of the acoustic emissions of the wrist joint (recorded at 150 Hz–20 kHz). Eight sensor positions were evaluated using uniaxial accelerometers as contact microphones that were fixed with double-sided tape. Nine audio features (zero-crossing rate, acoustic energy, spectral centroid, spectral spread, spectral flux, harmonic ratio, spectral crest, spectral decrease, and spectral slope) were used to obtain an average feature vector that was used for the calculation of ICCs. The suggested framework showed fair-to-high levels of repeatability with intrasession and intersession ICC values from 0.629 to 0.886 at three sensor locations. The authors reported that sensor locations (directly above areas of radius and ulna heads) with the highest repeatability (ICCs > 0.8) also showed a higher level of noise and motion artifact interference due to the skin motion relative to the underlying skeletal structure, while sensors placed further (3 cm) from the wrist joint had a higher signal-to-noise ratio due to reduced skin movement, but lower levels of intersession and intrasession repeatability (ICC > 0.6).

The outcomes of these two studies suggest that joint acoustic emissions are most likely to be reproducible in multiple recordings of the same subject; however, changes in sensor placement and fixation, motion artifacts and external noise due to straps/tape friction and cable movements can highly affect the quality of the recording. To address the above-mentioned issues, a novel motion artifact-resistant attachment of AE sensors and a robust data recording procedure were recently developed by the authors of this paper [22]. As both researchers and clinicians require evidence that the tests they use are able to provide valid and trustworthy results, this study aims to evaluate the inter-day and intrasession reliability of the improved and standardized methodological design of the joint AE recording procedure on a cohort of healthy young volunteers. In addition, the study permits the identification of the physical activities and AE signal features that yield the most reliable evaluations, hence facilitating the further development of AE monitoring and translation into clinical practice as an orthopedic diagnostic tool.

## 2. Materials and Methods

The study is presented in accordance with the Guidelines for Reporting Reliability and Agreement Studies (GRRAS) [23]. A convenience sample of eight healthy young volunteers was recruited for this study: four females, BMI: 24.07 (SD2.83), age: 33 (SD 5.26) years, with no record of major musculoskeletal, skin, or any other major disorder or injury. Sample size was chosen according to Walter et al. [24] with three measurement sessions per participant, a significance level of 0.05, and a power of 0.80, with the values of the reliability coefficients anticipated to be between 0.6 and 0.9.

### 2.1. Experimental Protocol

After enrolment, each participant was scheduled for three data collection sessions with an interval of 7.5 ± 5.1 days (minimum interval: 1 day; maximum interval: 16 days) between sessions. Sessions were arranged at a different time of the day and with different between-session intervals to emulate real-world clinical practice conditions, however, no significant change was expected in joint AEs as young and healthy volunteers were recruited. All the measurements were conducted by a single rater.

**Figure 3:**
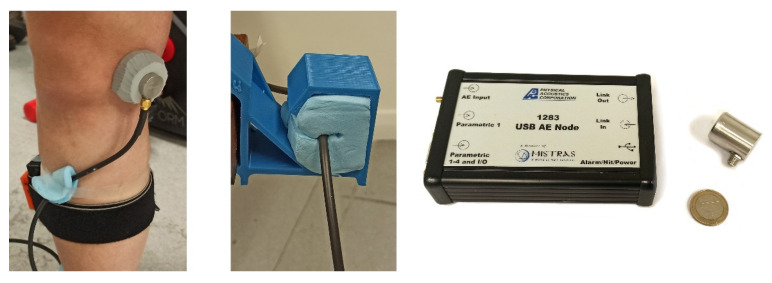
Testing setup: sensor fixation (**left**), cable fixation (**center**), USB AE Node monitoring system (**right**).

Knee-joint AE events were recorded by using a commercially available USB AE Node monitoring system (Mistras, Physical Acoustics) and a PK151 AE sensor (Figure 3, right) with an operational frequency range of 100–450 kHz and an integrated pre-amplifier [25], which can be found in Figure 1. The sensor weighs 51 g and has a height and a diameter of 27 mm and 20.6 mm, respectively. The choice of the sensor was based on commercially available ready-to-use low-noise compact sensors in the medium frequency range suitable for the recording of joint AEs [10]. While smaller and more lightweight sensors exist (e.g., Nano30, Physical Acoustics [26]), they are generally less sensitive (−72 dB Ref V/µbar for Nano30 vs. −36 dB for PK151) and their frequency response is generally higher than that which was previously used for joint AE monitoring [10].The selected sensor was attached to the right medial tibial condyle area [14] using ethylene-vinyl acetate (EVA) foam holders with a density of 100 kg/m^3^ and double-sided skin-safe tape (Arcos, transparent tape) (Figure 3, left). This configuration employing the high-density EVA foam holder with a diameter of 4.5 cm produced the most stable fixation, external noise isolation, and little to no noise during straight-leg motions during preliminary trials [27].The connecting cable was secured with a plastic holder with cross-linked polyethylene foam (25 kg/m^3^) isolation (Figure 3, center) and was additionally taped to the leg to reduce noise from unwanted cable movement. A further detailed description of the utilized motion artifact-resistant sensor attachment is available in [27].

The hit definition parameters were preset to the following values: PDT = 200 µs, HDT = 800 µs, HLT = 1000 µs, with a registration threshold equal to 32 dB (around 40 µV) based on the results of Shark et al. [28] and according to the previously used frequency range in the literature [10]. Inertial measurement unit (IMU) sensors (Xsens Technologies B.V., Enschede, Netherlands) were used to obtain the angular positions of the shank and the thigh of the participants, and the crank’s angle of the stationary bicycle during the cycling exercise. Two IMU sensors were placed on the thigh and shank according to the manufacturer’s guidelines, and the angular position of the sensors was set to zero during standing (straight leg) and when the pedal was at the lowest position during cycling before the start of the exercise. Each session was initiated by a short warm-up of 5 min of walking. Participants were then asked to perform ten repetitions for each of the following five exercises in a random order: straight-leg hip flexion/extension (SLF), straight-leg hip abduction/adduction (SLA), single-leg squats (SLS) and knee lifts (KL). These exercises were selected in order to capture straight-leg movements in both the sagittal (SLA) and frontal (SLF) planes, and knee flexion with (SLS) and without load (KL), thus facilitating the validation of the technique in different conditions and when different numbers of AE events are produced. Moreover, such exercises are frequently utilized in rehabilitation programmes in cases of post-injury, knee replacement, and osteoarthritis [29,30,31]. Knowing which exercises produce valid AE measures has the potential to improve not only AE diagnostic value, but also rehabilitation programmes by using AE to evaluate the patient’s progression in a more objective manner.

Only a small number of hits (or none) were expected from the knee during the SLA and SLF tasks, as these exercises do not involve any bending or loading of the knee. Therefore, these two exercises can be used as an indicator of a flawed sensor attachment or inappropriate positioning of the transducer. In cases of recordings showing significantly elevated noise (i.e., more than five hits per repetition) during the SLA or SLF, the sensor was re-attached and the participant was asked to repeat all exercises. In contrast, SLS and KL were expected to show a higher number of AE events and a greater variability between participants due to the differences in joint loads (e.g., due to the variability of the participant’s weight) and the knee’s range of motion. To help participants execute exercises uniformly, a metronome with a tempo of 20 bpm was used.

It was demonstrated previously that a significant number of acoustic events in repetitive motions occur consistently at specific joint angles [32]. Therefore, to minimize potential variability in exercise execution between sessions, a constrained exercise (cycling) was also included in the trials. Participants were asked to cycle on a stationary bike with two cadences and two resistance braking settings. Stable cadence (30 and 60 rpm) was achieved by using a metronome and a cadence sensor connected to a smartphone to provide audible and visual feedback to the participant. The lowest (L) and highest (H) available resistance settings on the stationary exercise bike (121Perform, CardioForm) were selected to attain controlled joint-loading conditions. The resulting four cycling modes (L30, H30, L60 and H60) were recorded for 1 min each in a randomized order. Before the start of the recording, participants were given some time to familiarize themselves with the cycling exercise tempo.

### 2.2. Data Processing

**Figure 4:**
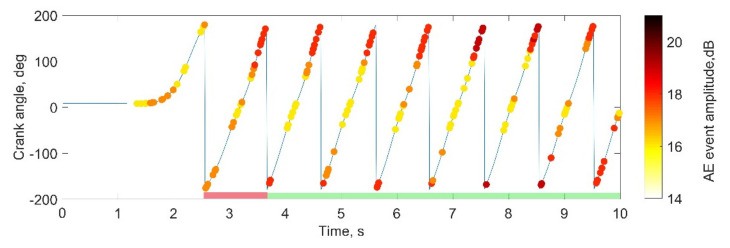
Synchronized bike crank orientation and AE hits recording. Repetitions included in analysis are indicated with a green line.

AEwin software (Mistras, Physical Acoustic) was used to obtain and export AE recordings in an ASCII format. Recordings, along with the IMU sensor orientation, were further analyzed in MATLAB (Mathworks). Firstly, AE recordings were time synchronized with motion data, and then IMU orientation was used to segment recorded signals into repetitions, with one rotation corresponding to one repetition for the cycling exercises. Synchronization of the AE and IMU signals allowed allocating hits to the respective angles of the joint and calculating the number of hits within a single repetition. An example of the synchronized signal is presented in Figure 4, with each dot corresponding to a recorded AE event and repetitions corresponding to the change of the crank angle between −180 and 180°; additionally, the amplitude of AE hit is color-indicated.

For the non-cycling exercises, eight repetitions were included, with the first and last repetitions being discarded due to additional noise and/or compromised execution in several records (e.g., noise from foot contact with the ground during the initiation and termination of the exercise). For cycling, only repetitions (rotations) with a specific duration were included with an accepted time range of 1.8 to 2.2 and 0.9 to 1.1 s (i.e., the assigned cadence with a 10% tolerance) for the 30 and 60 rpm cadences respectively (e.g., in Figure 4, the first repetition is excluded). The first 40 rotations that met these timing requirements were chosen for the 60 rpm trials, and likewise, the first 20 rotations for the 30 rpm trials.

The parameters presented in Table 1 were exported from the AEwin software and were included in the reliability evaluation. Measurements and respective units for the extracted parameters are specific to the hardware (Mistras system) and software (AEwin) that were used during the AE recordings [33]. The mean number of hits per repetition was calculated for each exercise. For the remainder of the hit parameters, mean values were obtained from all the detected AE events in the included repetitions.

The full tables containing means and standard deviations, median values, interquartile ranges, as well as CVs for each exercise can be found in Supplementary Materials.

### 2.3. Statistical Analysis

IBM SPSS Statistics was used to perform the statistical analysis. For the evaluation of the test-retest (inter-day) reliability, the ICC was used, an appropriate measure of reliability assessment in clinical applications [34]. ICC values were computed using an average measurement, absolute agreement, a two-way mixed-effects model, and an 95% confidence interval (CI) [34,35]. ICC values of less than 0.5 were regarded as indications of poor reliability, those between 0.5 and 0.75 as moderate reliability, between 0.75 and 0.9 as good reliability, and those greater than 0.90 as evidence of excellent reliability [35]. Coefficient of variation (CV) was also used to assess the dispersion of measurements around their mean between participants. Exercises involving straight-leg movements (SLA, SLF) were excluded from a further reliability assessment as the AE hits that were captured during these recordings can be attributed to either noise or acoustic AE events originated from the hip joint during movement [27].

In this study, the intrasession reliability was defined as the repetition-to-repetition reliability within the same exercise and session. Intrasession reliability was evaluated using the number of AE hits, as this parameter showed relatively high ICC in inter-day assessments (Table 2). Additionally, the number of AE hits relates to the previously reported test-retest reliability of joint sounds amplitude [21], thus allowing further comparisons with our findings. Eight repetitions for SLS and KL and the first twenty cycling rotations with the desired duration (1 and 2 s for the 60 and 30 rpm with 10% tolerance, respectively) were included in the intrasession assessment. The ICC was computed using a single measurement, an absolute agreement, a two-way mixed-effects model and a 95% confidence interval [35]. CVs were calculated for each participant for all sessions.

## 3. Results

### 3.1. Inter-Day Test-Retest Reliability

Inter-day test-retest ICCs ranged from poor (0) to good (0.901) for the investigated AE event parameters and exercises (Appendix A, Table A1). Several parameters showed low reliability (Table 2, Appendix A, Table A1) with some instances displaying a higher value of mean-square values within a subject than the corresponding between subjects values, resulting in a bad estimate of reliability and negative ICCs [36]; such ICC values are presented as zeros [37].

The mean number of hits per repetition, rise time, duration, and reverberation frequency displayed ICCs of more than 0.75 for at least half of the included exercises (Table 2). For the cycling datasets (L30, H30, L60, H60), the ICC for all included parameters ranged from 0.538 to 0.901, while KL and SLS generally showed poor reliability (less than 0.5), with the exception of the number of hits per repetition for the SLS (ICC = 0.716). CV values for all exercises ranged from 0.212 to 1.298 for the number of hits per repetition, rise time, duration, and reverberation frequency between participants during each session: CV1, CV2 and CV3. Table A1. Appendix A contains ICCs and CV values for all the investigated AE event parameters (Table A1).

### 3.2. Intrasession Reliability

Intrasession ICCs (repetition-to-repetition) ranged from 0.903 to 0.984 for the cycling exercises, and from 0.600 to 0.901 for the SLS and KL activities (Table 3). CV values for the cycling exercises were generally lower (0.074–0.708) than for the KL (0.127–1.512) and SLS (0.123–1.532), apart from one record during the L30 setting: participant 4, session 3.

### 3.3. AE Events Parameters

**Figure 5:**
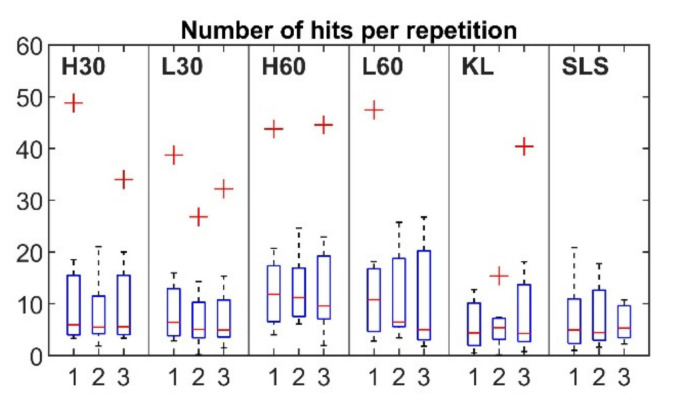
Box plot of number of hits per repetition for three sessions. Outliers are indicated by “+”.

**Figure 6:**
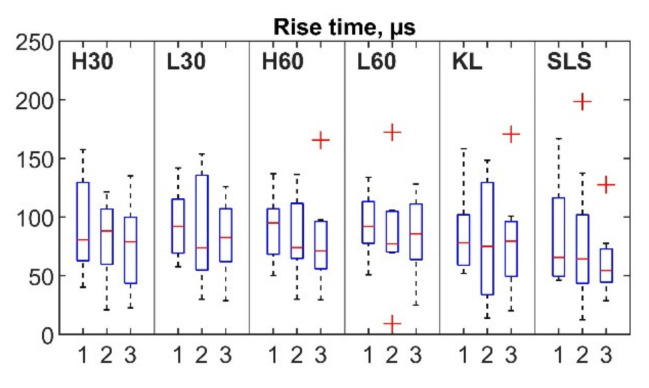
Box plot of AE hit rise time for three sessions. Outliers are indicated by “+”.

**Figure 7:**
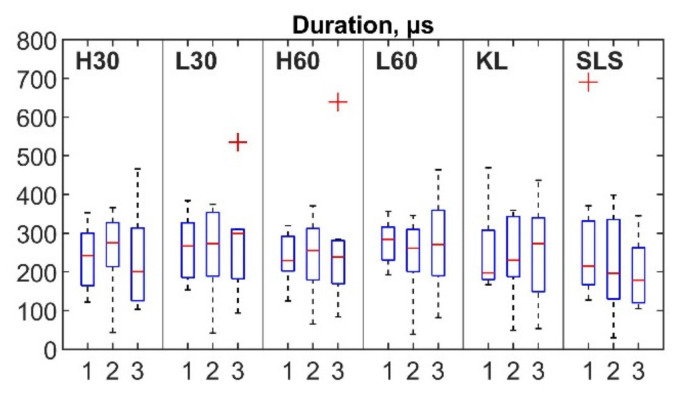
Box plots of AE hit duration for three sessions. Outliers are indicated by “+”.

**Figure 8:**
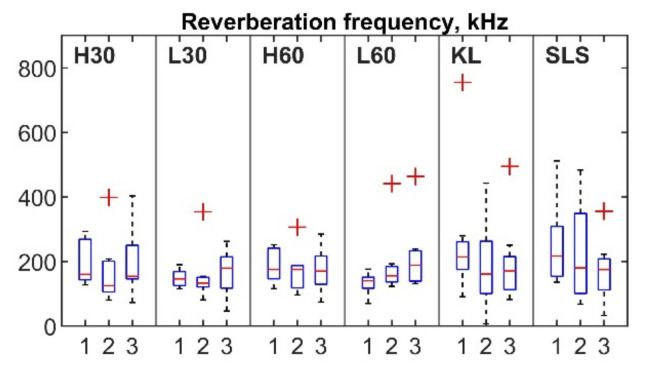
Box plots of AE hit reverberation frequency for three sessions. Outliers are indicated by “+”.

For each session (Figure 5, horizontal axis: 1, 2 and 3 sessions), the mean number of hits per repetition (vertical axis) ranged from 8.16 to 14.79 for the cycling, from 5.76 to 10.29 for the KL and from 6.22 to 7.42 for the SLS. Outliers were detected for all exercises apart from the SLS. Nine out of ten outliers were observed for the same two participants (five for participant 3 and four for participant 6, with four and three outliers respectively observed within the same session). The mean rise time for the first, second and third sessions was in the range of 75.34–94.1 µs for the cycling and 66.29–86.07 µs for the SLS and KL tasks (Figure 6). Six outliers from the records of four participants (1, 4, 5, 8) were also detected. The mean duration values ranged between 231.04 and 276.42 µs for the cycling and 241.14 and 251.73 µs for the KL; and 205.1 and 280.5 µs for the SLS (Figure 7). Only two outliers were detected (participant 5, session 3) for the mean hit duration parameter. Finally, mean reverberation frequency values ranged from 132.93 to 250.94 kHz for the included exercises (Figure 8). Eight outliers were also detected, with six being attributed to the same participant during the same session (participant 4, session 2).

## 4. Discussion

### 4.1. Reliability of Knee AE Monitoring

The results of the intrasession reliability are overall aligned with previously published findings [21], where AE measurements that were conducted without replacement of the microphone on the same day also showed moderate to excellent (ICCs of 0.73–0.95) reliability for the sit-to-stand exercise. In this study, slightly lower values (ICCs of 0.600–0.901) were obtained during the SLS and KL, while all cycling exercises showed better results with ICCs of more than 0.9. It can also be noted that by imposing more movement constraints on an exercise, better reliability of the measurement can be achieved. This was also confirmed by the generally lower levels of variability (CV values below 0.7 for the majority of the participants) reported for the cycling (Table 3). Additionally, for the cycling, only the repetitions that followed a cadence with a 10% tolerance were included in the analysis, thus further increasing the exercise’s execution consistency. These findings, along with the work published by Teague et al. [32] that reported consistency of AE events at specific joint angles, suggest that keeping the angle and motion trajectory consistent during AE recordings can significantly improve the reliability of all measurements.

The use of cycling adopted in this study is a novel approach, and to the best of the authors’ knowledge, it has not been previously reported as a diagnostic exercise in joint AE monitoring. Cycling has the additional advantage of standardized loading of the knee joint and eliminating the impact of the bodyweight, which has previously been found to influence the number of recorded hits [17]. In this way, a higher diagnostic value of AE monitoring can be achieved, as the recorded AE events would mainly be attributed to the physiological and pathological changes in the joint, rather than the loading from the participant’s weight, which is especially relevant to obese people. For the chosen parameters, neither load (resistance) nor cadence showed any discernible benefit in the inter-day assessment. This is most likely due to the bike resistance-braking levels selected being too low to have any significant effect on the registered joint AE; the levels are also significantly lower than the regular joint load in daily life during walking and could be safely increased. Therefore, higher resistance levels during measurements might provide greater diagnostic value, as a larger number of AE events would be registered, possibly indicating more subtle differences.

In this study, it was shown that the inter-day ICCs were higher than the values that were previously reported (0.24 to 0.33 for the tibial microphone position and 0 to 0.82 for the patellar microphone position) [21], indicating the potential of the employed method for improving the quality of the AE monitoring. While results from the above-mentioned study [21] cannot be directly compared with our findings, since different parameters and frequency ranges for the AE signals acquisition were used, it can be noted that the acquisition method and the constrained exercises in conjunction with a motion artifact-resistant sensor mounting in the present study improve the overall reliability of the measurements, with ICCs ranging from 0.538 to 0.901. Additionally, using straight-leg movement as a reference measurement (baseline recordings with little to no AE events) to identify compromised sensor fixation ensures that preventable motion artifacts and background noises (e.g., due to excessive cable swinging or unsticking of the tape) are avoided.

**Figure 9:**
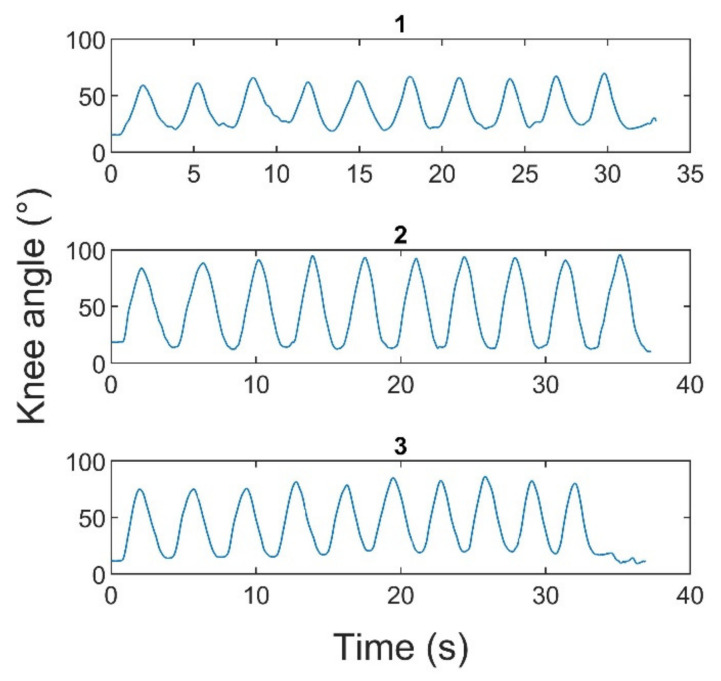
Example of difference in the knee angle between trials: KL, participant 4.

Poor inter-day reliability of the KL and SLS tasks might be attributed to the difference in exercise execution between sessions. This can be better illustrated with the example of participant 4 (Figure 9), whose knee flexion/extension angles during KL were relatively similar within the same session, with low CV values of 0.0826, 0.027, and 0.037 for the three recordings, respectively; however, dissimilar angle values were observed during different sessions (1, 2, 3) with a difference of more than 30 degrees: mean values and standard deviations of 43.33 ± 3.58°, 79.10 ± 2.13°, and 63.42 ± 2.31° for the first, second, and third sessions, respectively. Moreover, the detected joint AEs might potentially be affected by the changes in the pattern of exercise execution. For instance, phase durations of an exercise, such as lifting and lowering of the lower leg, can differ despite the use of a metronome. For example, for the same participant, the mean durations of the leg lowering phase during the KL for the first and second sessions were equal to 1.58 (±0.52) and 2.15 (±0.29) seconds, respectively, while the mean duration of leg lifting was equal to 1.41 (±0.23) and 1.52 (±0.34) seconds, respectively.

Although a number of AE event parameters were evaluated, only four (the number of hits per repetition, duration, rise time, and reverberation frequency) showed ICCs of more than 0.75 for at least three exercises. These parameters should be further considered in the development of AE joint monitoring as they showed acceptable levels of consistency. Additionally, even though mean values did not differ significantly between sessions for the majority of the registered parameters (Figure 5, Figure 6, Figure 7 and Figure 8), a number of outliers were detected. Reverberation frequency and hits per repetition showed the highest number of outliers. Notably, the majority of the outliers were detected within the same session for the same participant, indicating a possible flawed procedure, or, less likely, a high individual variability of the knee joint AEs.

### 4.2. Limitations of the Present Study and Future Work

While the findings described are potentially applicable to a wide range of AE monitoring applications for joint assessment, it should be taken into account that measurements were taken using custom sensor-mounting techniques that were previously optimized by the authors [27] in a cohort of healthy young volunteers. Additionally, an AE sensor with specific characteristics and explicit hit-detection parameters (as outlined in Section 2.1) was used to record and define AE events. Additional investigation of software and hardware configuration during recording could potentially improve the overall reliability of the method.

The reliability coefficients were lower than anticipated for some of the parameters and/or exercises, resulting in wide confidence interval bounds, thus a larger sample might be beneficial to reduce the uncertainty and further evaluate the reliability of the method in such cases.

The current study laid the groundwork for the further development of a reliable method of knee AE monitoring, including the recording approach, exercises, and AE parameters that produce the most reliable results. However, future work may include trials with a larger sample of healthy volunteers in different age groups, along with patients with specific joint conditions, to further assess the reliability of using AE monitoring for the assessment of the knee or hip in multiple cohorts. Moreover, investigation of the additional factors that can contribute to differences in inter-day measurements, such as the effects of perspiration on the sensor fixation or recent intense physical activity on joint AEs, as well as optimization of the preset hit definition parameters for a specific joint and application, may further improve the reliability of the recordings.

## 5. Conclusions

This study presents an AE monitoring inter-day and intrasession reliability evaluation using recordings from a set of healthy young volunteers with no history of knee joint injuries or degenerative diseases over a series of exercises. The results showed that exercises with imposed constraints, such as cycling, should be preferably used in the evaluation of knee joint acoustic emissions, as movement constraints and standardized joint-loading conditions ensure repeatability in the detection of the joint’s AE events. Moreover, using the presented recording procedure, featuring reference exercises (a straight-leg movement without load) and foam noise isolation can be helpful in ameliorating the effect of motion artifacts, increasing the consistency of the measurements in comparison with results previously reported in the literature. From the range of the investigated AE parameters, number of hits per repetition, duration, and rise time of the hit showed better reliability and could potentially be used for further development of a reliable knee joint assessment method with AE monitoring.

AE monitoring is shown to be a technique that is highly prone to noise. However, with appropriate measures to ensure minimization of motion artifacts and consistency in joint movements, the overall reliability of the method can be improved, thus allowing further investigation of possible AE biomarkers for a range of joint conditions, the design of new sensors, and the refinement and standardization of AE monitoring procedures, that may bring joint AE monitoring closer to clinical practice.

## Figures and Tables

**Table 1:** AE Event (Hit) Parameters.

Parameter	Definition	Measurement Unit
Hits number	Number of the detected AE hits	-
Rise time	Time between detected AE hit start and its peak amplitude	µs
Counts	Number of an AE signal excursions over the threshold in one hit	-
Duration	Time from the first threshold crossing of the hit to the last	µs
Amplitude	Maximum AE signal excursion during a hit	dB
Counts to peak	Number of an AE signal excursions over the threshold between AE hit start and its peak amplitude	-
Signal strength	Integral of the rectified voltage signal over the duration of an AE hit	pV-s
Absolute (true) energy	Integral of the squared voltage signal divided by the reference resistance over the duration of an AE hit	attoJoules
Average frequency ^1^	Average frequency over the entire AE hit	kHz
Reverberation frequency ^1^	Average frequency of AE hit after the peak	kHz

^1^ Frequency parameters are calculated by AEwin software using previously obtained hit parameters, e.g., duration and counts. Measurement units are presented as in the exported AE recordings.

**Table 2:** Inter-day test-retest reliability: intraclass correlation coefficients (ICC) with 95% confidence intervals and coefficients of variation between subjects for three sessions (CV1, CV2, CV3).

Exercise ^1^	ICC	Lower Bound (95% CI)	Upper Bound (95% CI)	CV1	CV2	CV3
Hits per repetition: mean value						
L30	0.771	0.205	0.951	1.093	1.069	1.137
H30	0.694	0	0.934	1.120	0.773	0.992
L60	0.901	0.681	0.978	1.015	0.798	0.980
H60	0.765	0.163	0.950	0.861	0.528	0.919
KL	0.381	0	0.866	0.843	0.784	1.298
SLS	0.716	0	0.940	0.926	0.811	0.544
Rise time (µs): mean value						
L30	0.828	0.422	0.963	0.326	0.523	0.389
H30	0.844	0.503	0.965	0.441	0.417	0.510
L60	0.538	0	0.903	0.292	0.533	0.402
H60	0.785	0.267	0.954	0.307	0.409	0.514
KL	0.073	0	0.790	0.417	0.658	0.562
SLS	0.200	0	0.832	0.706	0.763	0.487
Duration (µs): mean value						
L30	0.810	0.332	0.959	0.319	0.454	0.482
H30	0.794	0.279	0.956	0.350	0.406	0.556
L60	0.856	0.533	0.968	0.212	0.407	0.456
H60	0.728	0.025	0.942	0.270	0.406	0.637
KL	0	0	0.526	0.419	0.444	0.521
SLS	0.513	0	0.893	0.563	0.581	0.444
Reverberation Frequency (kHz): mean value						
L30	0.712	0	0.938	0.182	0.534	0.420
H30	0.827	0.426	0.962	0.357	0.616	0.517
L60	0.567	0	0.899	0.243	0.550	0.512
H60	0.696	0	0.935	0.280	0.387	0.384
KL	0	0	0.710	0.764	0.750	0.671
SLS	0.102	0	0.807	0.659	0.667	0.555

^1^ L30—low load, 30 rpm cycling, H30—high load, 30 rpm cycling, L60—low load, 60 rpm cycling, H60—high load, 60 rpm cadence cycling, KL—knee lift, SLS—single-leg squat.

**Table 3:** Inrasession- test-retest reliability: intraclass correlation coefficients (ICC) with 95% confidence intervals and coefficients of variation between subjects for three sessions (CV1, CV2, CV3).

Exercise ^1^	ICC	Lower Bound (95% CI)	Upper Bound (95% CI)	CV Range
Session 1				
L30	0.974	0.941	0.994	0.110–0.336
H30	0.984	0.963	0.996	0.074–0.347
L60	0.984	0.963	0.996	0.065–0.271
H60	0.971	0.934	0.993	0.077–0.323
KL	0.760	0.541	0.933	0.217–1.512
SLS	0.795	0.593	0.944	0.146–1.532
Session 2				
L30	0.903	0.796	0.975	0.134–0.371
H30	0.942	0.872	0.986	0.097–0.611
L60	0.964	0.918	0.991	0.082–0.582
H60	0.920	0.828	0.980	0.086–0.601
KL	0.725	0.492	0.921	0.204 ^2^–0.672
SLS	0.884	0.746	0.971	0.123–1.088
Session 3				
L30	0.924	0.835	0.981	0.152–1.593
H30	0.949	0.887	0.987	0.089–0.479
L60	0.966	0.923	0.992	0.087–0.544
H60	0.966	0.923	0.992	0.072–0.708
KL	0.901	0.777	0.975	0.127–0.943
SLS	0.600	0.343	0.872	0.247–1.000

^1^ L30—low load, 30 rpm cycling, H30—high load, 30 rpm cycling, L60—low load, 60 rpm cycling, H60—high load, 60 rpm cadence cycling, KL—knee lift, SLS—single-leg squat. ^2^ KL record for participant 4 did not contain any hits in the majority of repetitions and respective CV value (2.823) was excluded as mean hits number is close to zero (0.125).

## Data Availability

The data presented in this study are available on request from the corresponding author. The data are not publicly available due to ethical restrictions.

## Supplementary Materials

The following supporting information can be downloaded at: https://www.mdpi.com/article/10.3390/s22239027/s1, full tables presenting means and standard deviations, median values, interquartile ranges, as well as CVs for each exercise (L30.xlsx, H30.xlsx, L60.xlsx, L60.xlsx, KL.xlsx, SLS.xlsx, SLA.xlsx, SLF.xlsx).

## Appendix A

**Table A1:** Inter-day test-retest reliability: intraclass correlation coefficients (ICC) with 95% confidence intervals and coefficients of variation between subjects for three sessions (CV1, CV2, CV3).

Exercise ^1^	ICC	LOWER Bound (95% CI)	Upper Bound (95% CI)	CV1	CV2	CV3
Counts: mean value						
L30	0.383	0	0.867	0.342	0.333	0.715
H30	0.752	0.167	0.946	0.378	0.576	0.679
L60	0.532	0	0.897	0.239	0.675	0.838
H60	0.581	0	0.910	0.313	0.527	0.969
KL	0	0	0.326	0.615	0.410	0.454
SLS	0.474	0	0.884	0.828	0.550	0.563
Amplitude (dB): mean value						
L30	0.309	0	0.860	0.024	0.034	0.060
H30	0.783	0.213	0.954	0.028	0.037	0.029
L60	0.128	0	0.826	0.015	0.023	0.058
H60	0.256	0	0.846	0.022	0.031	0.072
KL	0	0	0.608	0.046	0.036	0.040
SLS	0.532	0	0.899	0.127	0.051	0.070
Counts to peak: mean value						
L30	0.495	0	0.892	0.301	0.409	0.604
H30	0	0	0.657	0.380	0.386	0.557
L60	0.532	0	0.897	0.215	0.685	0.777
H60	0.656	0	0.926	0.290	0.432	0.827
KL	0	0	0	0.543	0.413	0.487
SLS	0.323	0	0.855	0.532	0.458	0.381
Signal Strength (pV-s): mean value						
L30	0.770	0.191	0.951	0.403	0.506	0.580
H30	0	0	0.762	0.337	0.450	0.608
L60	0.684	0	0.933	0.273	0.488	0.529
H60	0.539	0	0.904	0.301	0.456	1.020
KL	0	0	0.613	0.556	0.501	0.727
SLS	0.084	0	0.799	2.370	0.735	0.486
Absolute Energy (attoJoules): mean value						
L30	0.741	0.165	0.943	1.747	0.640	1.227
H30	0.734	0.158	0.941	0.349	0.803	2.560
L60	0	0	0.528	0.798	0.745	0.626
H60	0.409	0	0.875	1.173	1.009	1.438
KL	0	0	0.570	1.281	0.659	1.276
SLS	0.001	0	0.781	2.722	0.944	0.575
Average Frequency (kHz): mean value						
L30	0.555	0	0.907	0.179	0.781	0.426
H30	0.720	0.112	0.938	0.319	0.691	0.522
L60	0.674	0.024	0.926	0.216	0.693	0.575
H60	0.766	0.152	0.950	0.277	0.463	0.426
KL	0.036	0	0.798	0.940	0.844	0.913
SLS	0	0	0.694	0.606	0.659	0.728

^1^ L30—low load, 30 rpm cycling, H30—high load, 30 rpm cycling, L60—low load, 60 rpm cycling, H60—high load, 60 rpm cadence cycling, KL—knee lift, SLS—single-leg squat
